# The Expression of MicroRNA-21 in Bone Marrow Fluid is an Indicator of Hematological Disorders and Mortality

**DOI:** 10.31557/APJCP.2020.21.10.2817

**Published:** 2020-10

**Authors:** Wei-Ting Chang, Tzu-Ling Huang, Zhih-Cherng Chen, Yin-Hsun Feng

**Affiliations:** 1 *Division of Cardiology, Department of Internal Medicine, Chi-Mei Medical Center, Tainan, Taiwan.*; 2 *Department of Biotechnology, Southern Taiwan University of Science and Technology, Tainan, Taiwan. *; 3 *Institute of Clinical Medicine, College of Medicine, National Cheng Kung University, Tainan, Taiwan. *; 4 *Department of Pharmacy, Chia Nan University of Pharmacy and Science, Tainan, Taiwan. *; 5 *Division of Hematology, Department of Internal Medicine, Chi-Mei Medical Center, Tainan, Taiwan. *; 6 *Department of Nursing, College of Medicine and Life Science, Chung-Hwa University of Medical Technology, Tainan, Taiwan. *

**Keywords:** U6, spiked-in RNA, miR-21, bone marrow fluid, hematological disorders

## Abstract

**Objective:**

Bone marrow fluid (BMF) consists of various components that establishes a microenvironment for cell differentiation and remodeling. MicroRNA-21 (miR-21) levels have recently emerged as novel biomarkers for different diseases. However, the conventional RNU6B (U6), used as the reference for intracellular miRNA, may not be appropriate for the normalization of circulating miRNAs.

**Methods:**

We measured the levels of U6, spiked-in RNA, and miR-21 in the BMF of 13 healthy controls and 37 patients with hematological disorders to investigate the reliability of either U6 or spike-in RNA as an endogenous reference and also to study the correlation between miR-21, hematological disorders and mortality.

**Results:**

Notably, the levels of U6 demonstrated a high variability in BMF of healthy controls and patients. In contrast, the levels of spiked-in RNA displayed a significantly higher stability in both cohorts. Compared with controls, the levels of miR-21 were significantly upregulated in BMF of patients with leukemia but not lymphoma. Also, using 21 as the cut-off value of miR-21, it differentiated the mortality of patients with hematologic disorders.

**Conclusions:**

Collectively, using spiked-in RNA as a reference the upregulated miR-21 levels in BMF could be an indicator of the diagnosis of leukemia and a predictor of mortality.

## Introduction

Bone marrow, a soft tissue, is present in the central hollow part of large bones, where the subtle microenvironment regulates the differentiation of hematopoietic stem cells into various blood components (Smith and Calvi, 2013). In clinical aspects, bone marrow aspiration and/or biopsy examination are conducted in case of an abnormal blood test, through which the physician can diagnose the progression of a certain disease or the response to a treatment (Malempati et al., 2009). An extract of supernatant from the bone marrow known as “bone marrow fluid (BMF),” which establishes a microenvironment for cell differentiation and invasion, has been underscored today (Hu et al., 2011). However, the regulatory mechanism is largely unknown.

MicroRNAs (miRs), of which the mature form is composed of approximately 20–22 nucleotides, are noncoding RNAs (Griffiths-Jones et al., 2006). In general, miRs regulate the mRNA expression by repressing mRNA translation and enhancing its degradation (Griffiths-Jones et al., 2006). Among them, miR-21, a conserved gene, is located on chromosome 17q23.2 of Homo sapiens and is an oncomiR causing numerous types of cancers and malignancies by aberration of the fine-tuned system of apoptosis and proliferation (Feng et al., 2011). A previous study showed that miR-21 is a widely expressed miR in hematopoietic cells (Bhagat et al., 2013). In addition, increasing evidence has demonstrated the correlation between miR-21 and hematological malignancies, including diffuse large B-cell lymphoma, natural killer cell leukemia, acute myeloid leukemia, and so on (Feng and Tsao, 2016). However, despite increasing research involving the identification of circulating miRs from plasma, serum, urine, and saliva, there exists a challenge in choosing a reliable endogenous reference since the stability of traditional RNU6B (U6) expression varies in different diseases (Benz et al., 2013). Therefore, the present study was conducted to explore the relationship between miR-21, BMF, and hematological disorders, including leukemia, lymphoma, and myelodysplastic syndrome (MDS). In addition, the current research compared the variability of the endogenous RNA gene U6 with the exogenous reference gene spike-in UniSp6 to choose a reliable reference gene.

## Materials and Methods

*Bone marrow fluid (BMF)*

From January 2013 to July 2018, we prospectively enrolled 40 patients receiving the first time of bone marrows studies in Chi-Mei Medical Center. Fifteen ml of bone marrow was aspirated and all enrolled patients were naïve from treatment. The definite diagnosis was made by pathologist according to the clinical practice guideline (Ghielmini et al., 2013). The informed consent was obtained from each patient and the study was conducted according to the recommendations of the Declaration of 1964 Helsinki on Biomedical Research involving human subjects. The protocols and procedures of the study have been approved by the Institutional Review Board of Chi-Mei Medical Center (CV code: 10406-E01). Bone marrows were obtained from four groups of patients who were categorized into the corresponding groups based on their clinical data as follows: (1) patients who were not suffering from hematological tumors were classified as normal controls, (2) patients with lymphoma, (3) patients with leukemia, and (4) those diagnosed with MDS. Fresh harvested bone marrow was centrifuged at 3,000 rpm at 4°C for 15 min, and then the supernatant was transferred to a clean Eppendorf tube for storage till further investigations. All methods were performed in accordance with the relevant guidelines. In addition, the clinical information including survivals were collected from the medical records post the enrollment of patients. 

*Quantitative real-time PCR (q-PCR)*

To conduct q-PCR, ExiLent SYBR Green master mix (Exiqon, 203421) was used, and the cDNA from reverse transcription was diluted 10 times. According to the manufacturer’s instruction, every reaction contained 5 µl of PCR master mix, 1 µl of primer, and 4 µl of diluted cDNA template. The conditions for q-PCR were as follows: (1) 10 min at 95°C for denaturation and polymerase activation, (2) 45 amplification cycles at 95°C for 10 s and 60°C for 1 min. The primers used included miR-21-5p (Exiqon, 204230), U6 (Exiqon, 203907), and UniSp6 (Exiqon, 203954).

*Statistics*

The variability of the endogenous gene U6 and the exogenous reference gene UniSp6 was analyzed by following the method of Fabian Benz et al., (2013). Briefly, the Ct difference to its median and the D2 value were computed, and a t-test was conducted to compare the differences between the two groups. The box plot displays the distribution of reference gene expression of the two groups, and the whisker plot represents the extension of the maximum and minimum values. Baseline characteristics of the four groups were initially compared by one-way ANOVA and then by logistic regression. The Kaplan–Meier method was used with a log-rank test to compare the survival rates between the strata.

**Figure 1:**
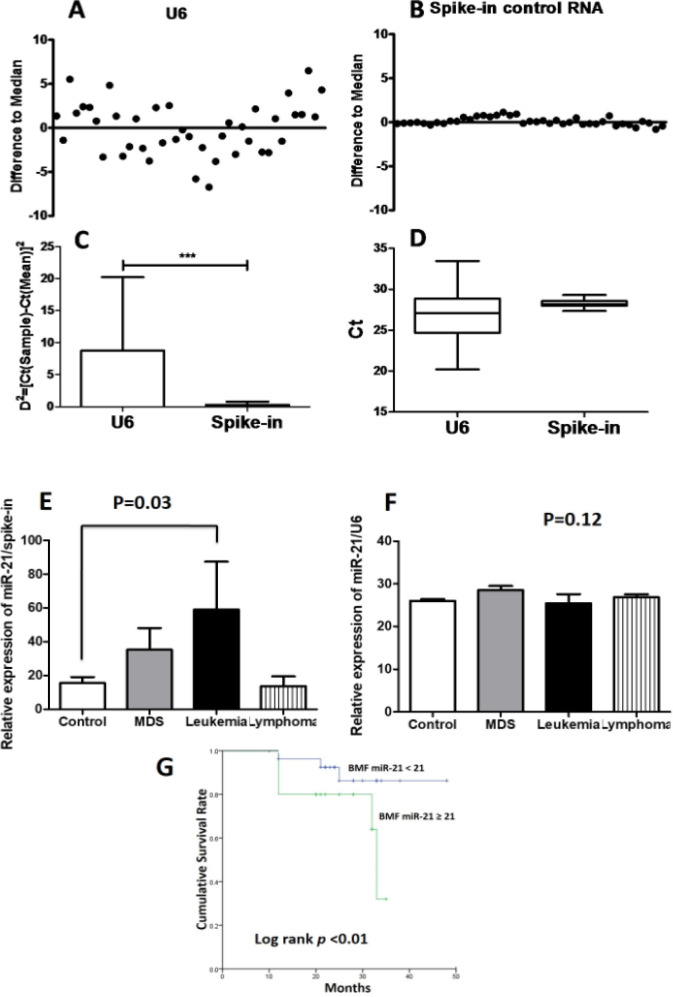
(A) RNU6B (U6) levels display high variability in the bone marrow fluid (BMF). (B) Levels of spiked-in RNA reveal a very low variability between the different samples. (C) The variability of U6 values and spike-in values was statistically compared, revealing a significantly higher variability in U6 values compared with that of spike-in. (D) Likewise, the raw U6 Ct values were compared with that of spike-in, revealing a higher stability in spike-in values. (D) Using the values of spike-in as references, the relative expression of miR-21 in BMF increased significantly in patients with leukemia compared with that in the control cohort. (E) Conversely, the expression of miR-21 to U6 ratios revealed no significant differences among groups. (F) In Kaplan-Meier analysis, patients with hematologic disorder and miR-21 relative expression above 21 representing a significantly increased mortality compared with those with miR-21 relative expression beneath 21 in five years. ***P<0.001

**Table 1:** The Baseline Characteristics and miR-21, U6 Expression in Bone Marrow Fluid (BMF) in the Control Cohort, Patients with MDS (Myelodysplastic Syndrome), Leukemia and Lymphoma

	Control (n=13)	MDS (n=15)	Leukemia (n=12)	Lymphoma (n=10)
Age (years)	58.3±20.0	64.6±18.4	31.3±20.6*	55±20.7
Male sex (%)	6 (46.1)	4 (26.6)	3 (25)	2 (20)
Body height (cm)	159.4±5.1	163.4±41.9	176±45.3	164.3±12.5
Body weight (kg)	52.6±8.2	59.6±14.9	79±19.7	59.4±15.1
Hypertension	3 (23)	1 (6.6)	0	1 (10)
Diabetes	2 (15.3)	2 (13.3)	0	0
Creatinine (mg/dl)	1.9±1.8	0.9±1.4	0.7±1.2	0.7±0.1
Glucose (mg/dl)	109.5±16.2	102±19.5	102±19.4	104.3±15.9
Calcium (mg/dl)	7.4±2.3	7.0±1.4	8.0±2.1	8.7±4.2
WBC (103/uL)	8.2±4.7	2.8±4.4	13.5±8.6*	14.7±2.6*
ANC	2216.7±744.1	1998.2±345.1	6097.5±860.6*	7373.8±762.5*
Hemoglobulin (g/dl)	10.5±2.6	8.7±3.2	11.3±3.3	9.9±2.7
Platelet (103/uL)	267.3±166.2	154.7±144.4	291.6±164.9	218.8±121.6
AST (IU/L)	21.4±6.9	27.5±7.6	131±30.2	21.5±6.0
ALT(IU/L)	27±3.5	23.6±3.6	36±21.4	21.2±4.3
LDH	166±21.8	162.5±41.2	285±32.8*	262.5±43.3*
miR-21 expression in BMF	18.7±8.4	32.6±18.2	63.7±22.2*	17.9±6.8
U6 expression in BMF	20.9±1.8	28.4±3.2	25.4±4.2	26.8±2.2

Data are expressed as mean ± SD, or median. *P <0.05, compared with the control; MDS, myelodysplastic syndrome; WBC, white blood cell; ANC, absolute neutrophil count; LDH, lactic dehydrogenase; AST, aspartate aminotransferase; ALT, alanine aminotransferase

## Results

*The stability of spiked-in RNA rather than U6 as a reference in BMF*

To test the reliability of U6, one of the most widely used references for intracellular miRNA, in the BMF, we measured the levels of U6, spiked-in RNA, and miR-21 in the BMF of 13 healthy controls and 37 patients with hematological disorders. The levels of U6 demonstrated a high variability between individuals in the BMF of healthy controls and patients with hematological disorders (Figure 1A). In contrast, the levels of spiked-in RNA displayed a significantly higher stability in both cohorts (Figure 1B). In addition, the comparison of variability between U6 and spike-in levels revealed a significantly higher variability in U6 levels compared with that in spike-in levels (Figure 1C). Similarly, compared with the raw U6 Ct levels, those of spike-in indicated a higher stability (Figure 1D).

*Baseline characteristics of patients with hematological disorders*

Among the 13 healthy controls and 37 patients with hematological disorders, including 15 with MDS, 12 with leukemia (5 patients with acute myeloid leukemia and 7 patients with chronic myeloid leukemia), and 10 with lymphoma, patients diagnosed with leukemia were younger in age, but there were no significant differences in sex, body weight, body height, and baseline liver and renal functions (Table 1). Compared with the control cohort, the levels of WBC and ANC were significantly elevated in patients with leukemia and lymphoma but slightly decreased in patients with MDS. In addition, the LDH levels increased in patients with leukemia and lymphoma. Regarding the expression of miRNAs in BMF, miR-21 was significantly upregulated in patients with leukemia but not in patients with lymphoma. Notably, the levels of U6 were slightly elevated in patients with hematological disorders. Using the levels of spike-in as a reference, the relative expression of miR-21 in BMF was found to be increased significantly in patients with leukemia compared with that in the control cohort, while the expression of miR-21 also increased in patients with MDS, though not significantly (Figure 1E). Conversely, the ratios of the expression of miR-21 to U6 levels revealed no significant differences among the groups (Figure 1F). These results indicated that U6 may not be an ideal endogenous reference in BMF.

*The upregulated miR-21 in BMF independently associated with the diagnosis of leukemia*

To further study the correlation between miR-21 and hematological disorders, parameters that significantly changed in patients diagnosed with leukemia were included in the logistic regression (Supplement Table 1). Interestingly, compared with age, serologic WBC, ANC, and LDH levels, only miR-21 independently correlated with the diagnosis of leukemia (odds ratio: 1.21, confidence interval: 1.01–1.47, p = 0.04).

*The increasing expression of miR-21 in BMF predicted the mortality in patients with hematologic disorder*

Therefore, we deﬁned the median value of relative miR-21 expression cut-off point as 21. Kaplan–Meier survival curves revealed that the patients with miR-21 expression above 21 had high mortality during the follow-up period (Figure 1G). Discussion

The primary findings of this study were that (1) compared with U6, spiked-in RNA is a more optimal reference in BMF, (2) the upregulated miR-21 in BMF is independently associated with the diagnosis of leukemia, and (3) Using the cut-off value as 21 of relative miR-21 expression we differentiated the mortality of patients with hematologic disorder. To the best of our knowledge, this study is the first to validate the reference value of U6 and the clinical impact of miR-21 in patients’ BMF.

In recent years, the expressions of miRNAs have been shown to associate with various types of diseases, including cancers (Feng et al., 2011; Feng and Tsao, 2016). Most of the studies used U6 for normalization (Benz et al., 2013). However, despite showing a consistent expression in different tissues and cells, these intracellular findings cannot be directly translated into the situation in body fluids such as serum and BMF (Xiang et al., 2014). In previous studies, serum levels of U6 displayed an enormous interindividual variability in healthy volunteers as well as in untreated mice (Benz et al., 2013; Xiang et al., 2014). Moreover, serum levels of U6 showed a disease-specific regulation pattern in the respective patient cohorts when compared with those in healthy volunteers (Benz et al., 2013). Therefore, a reliable reference for the circulating microRNAs is crucial, and in this study, we found that instead of U6, spiked-in control RNA demonstrated a very low variability between the different samples. Thus, it could be an alternative choice for normalization.

MiR-21 is one of the earliest identified cancer-promoting “oncomiRs,” targeting numerous tumor suppressor genes associated with proliferation, apoptosis, and invasion (Feng and Tsao, 2016; Yue et al., 2016). The regulation of miR-21 and its role in carcinogenesis have been extensively investigated (Yue et al., 2016). Recent studies have focused on the diagnostic and prognostic value of miR-21 as well as its implication in the drug resistance of human malignancies (Bhaga et al., 2013; Feng and Tsao, 2016). Yue et al., (2016) found that blocking miR-21 function greatly abolished the promoting effect on the migration ability of cancer cells, resulting in the regulation of tumor metastasis. Regarding the impact of miR-21 in hematological malignancies, Labib indicated that upregulation of miRNA-21 is a poor prognostic marker in patients with childhood B-cell acute lymphoblastic leukemia (Labib et al., 2017). Moreover, targeting miR-21 induces autophagy and chemosensitivity of leukemia cells (Seca et al., 2013). According to our findings, miR-21 was significantly upregulated in the BMF of patients with leukemia. Similarly, in the logistic regression, miR-21 was independently associated with the diagnosis of leukemia. Interestingly, in our study, the association of miR-21 expression in BMF was absent in lymphoma, although a previous study indicated that dysregulation of miR-21 is pivotal in the pathogenesis of aggressive transformed, high-grade, and refractory lymphomas (Gu et al., 2013). Driven by the difference between leukemia and lymphoma, we proposed that the blood-forming malignant disease was prone to release miRNAs in BMF than malignancy of the lymphoid tissue like lymphoma.

BMF consists of various components that establish a microenvironment for cell differentiation and remodeling, while the signal transducer and activator of transcription (STAT)-3-regulated metabolic pathway was observed to be associated with miR-21 gene expression in chronic lymphocytic leukemia cells (Rozovski et al., 2013; Yue et al., 2016). Among the metabolic components linking obesity and cancer, adipokines, especially adiponectin, leptin, and resistin, existing in BMF, are known to induce tumorigenesis (Kang et al., 2013). In vitro, adiponectin is able to inhibit the proliferation of myelomonocytic progenitor cells and modulate apoptosis in acute myelomonocytic leukemia cell lines (Yokota et al., 2000). In an ApcMin/+ mice study, a smaller tumor size was observed when the mice were injected with adiponectin (Otani et al., 2010). Our previous study also showed a lower adiponectin concentration in the BMF of patients with a hematological malignancy (Linet al., 2015). This implies that adiponectin may contribute toward tumor suppression in the bone marrow. Collectively, this finding initiates a future study focusing on the expression of miRs, which regulates the microenvironment of cell differentiation and invasion, in BMF. Further investigations are required to unveil the mechanism of miR-associated development of hematological malignancies and their links between clinical stages and outcomes. 
